# Spiked Helmet Sign: An Uncommon Electrocardiographic Marker

**DOI:** 10.31083/j.rcm2409272

**Published:** 2023-09-25

**Authors:** Guangqiang Wang, Shu Zhong, Hongxia Chu, Lin Zhong

**Affiliations:** ^1^Department of Cardiology, The Affiliated Yantai Yuhuangding Hospital of Qingdao University, 264000 Yantai, Shandong, China

**Keywords:** spiked helmet sign, electrocardiogram, critically ill, noncardiac causes, poor prognosis

## Abstract

The spiked helmet sign (SHS) is a rare electrocardiographic marker associated with an increased risk of lethal ventricular tachyarrhythmias and sudden cardiac death. To our knowledge, this is the first study aimed at reviewing recent research progress on this electrocardiogram (ECG) pattern to summarize its electrophysiological mechanisms, epidemiological features, clinical characteristics, and clinical significance. SHS formation is attributed to sympathetic hyperactivity, which mediates increased dispersion of ventricular repolarization, leading to marked QT prolongation and macroscopic T-wave alternans. This pattern can be observed in critically ill patients with cardiac or noncardiac conditions. In particular, immediate identification of this ECG abnormality is crucial in recognizing and treating noncardiac conditions in older male patients.

## 1. Introduction

**Fig. 1.**
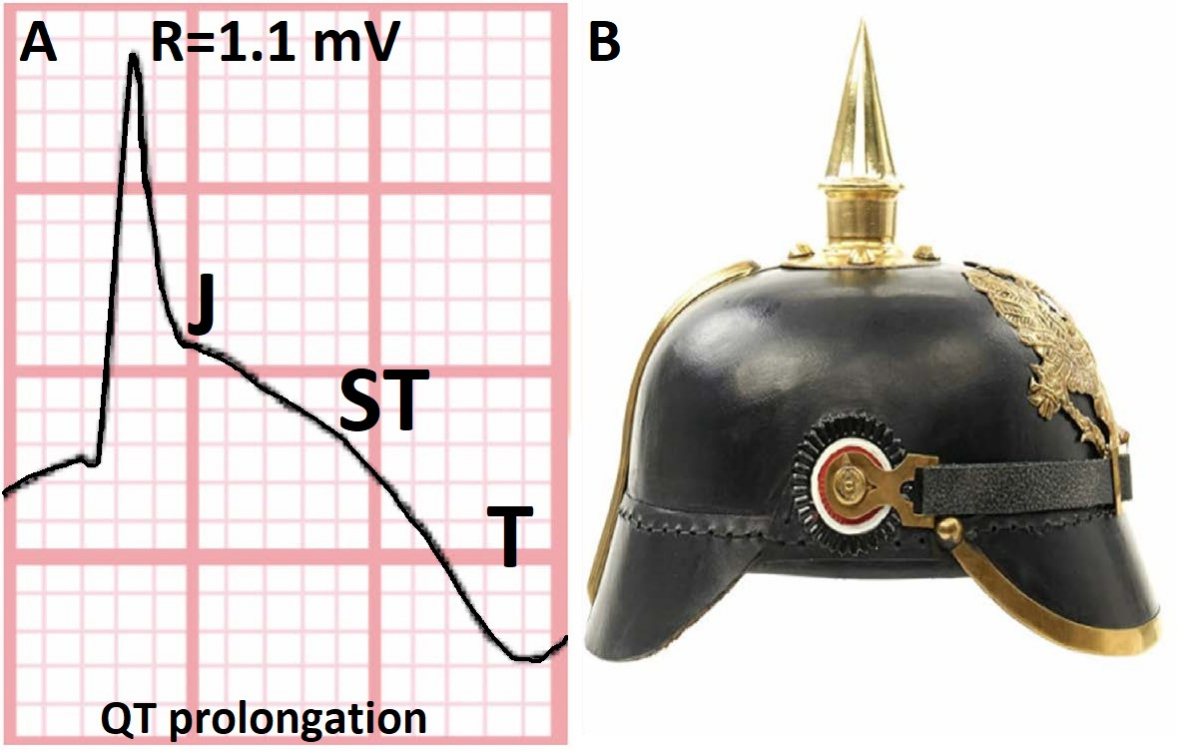
**Electrocardiographic representation of the spiked helmet sign (SHS) in QT prolongation (A) and its resemblance to the Pickelhaube German military helmet (B)**. (With permission from Crinion* et al*. [4]). The SHS QT motif, a rare electrocardiographic marker, is depicted in this figure. The distinctive curve observed in the electrocardiogram resembles the shape of the historical German military spiked helmet, known as the Pickelhaube. The SHS QT motif holds significance as it has been associated with poor outcomes within patient populations, making its identification and understanding crucial in clinical practice.

**Fig. 2.**
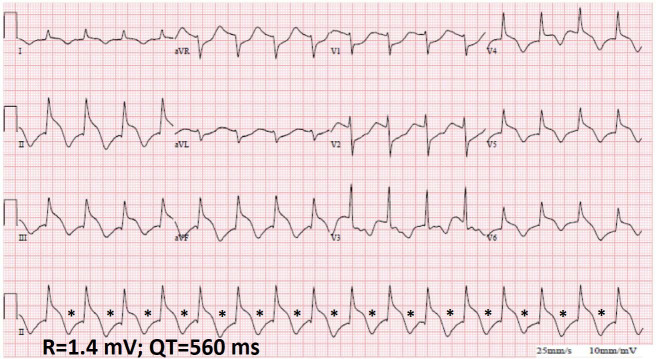
**A 12-lead electrocardiogram at the presentation showing inferior (II, III, aVF) and anterolateral (V4–V6) spiked helmet signs and macroscopic T-wave alternans**. The heart rate is regular, at 100 beats/min. Asterisks indicate the alternating T-waves. (With permission from Crinion* et al*. [4]).

The spiked helmet sign (SHS) a new electrocardiographic entity, was first described in 2011 by Littmann* et al*. [1]. This pattern is characterized by slurring or notching J-point elevation, subsequent downsloping ST-segment elevation, and wide T(U)-wave inversion in the inferior leads, suggesting a combination of J-point, elevated ST segment, and T-wave. The SHS width may correspond to the QT(U) interval. When the heart rate is sufficiently fast and the QT(U) is long enough, the inverted T(U)-wave reaches the subsequent QRS complex, and the upward and downward shifts of the electrocardiographic baseline reflect the ascending and descending limbs of the wide inverted T(U)-wave, respectively [2]. The distinguishing characteristic of this electrocardiogram (ECG) pattern is the late and giant T(U) waves of the preceding beat superimposed on the QRS complex [3]. This distinctive ECG pattern was named the Pickelhaube because it resembles the historical German military helmet, as depicted in Fig. 1 [4]. SHS was originally described in the inferior leads with subsequent publications reporting its presence in the anterior or lateral leads. Macroscopic T-wave alternans (TWA) refers to the beat-to-beat alternation of the amplitude or polarity of the T-waveform. Conventionally, TWA is considered an ominous sign of electrical instability and precedes fatal ventricular arrhythmias, especially imminent torsade de pointes (TdP). It is frequently associated with prolonged QT intervals. Additionally, macroscopic TWA and prolonged QT intervals are two crucial features of SHS on the ECG (Fig. 2) [4]. SHS has been reported in patients with severe disorders and major traumas of the brain, heart, lungs, or abdomen, as well as in cases of sepsis or thoracoabdominal aortic dissection [5]. SHS is a rare electrocardiographic marker associated with impending death during critical illnesses, particularly noncardiac illnesses [6]. This review presents the first comprehensive summary of the electrophysiological mechanisms, epidemiological features, and clinical implications of this ECG pattern.

## 2. Pathophysiology

In a typical ECG, the formation of the J point at the beginning of the ST segment is caused by early repolarization (Phase 1) in the major myocardial cells. The ST segment, appearing as an isoelectric horizontal line at the “baseline” in the ECG, represents the plateau phase (Phase 2) of the action potential (AP). T-wave duration ends when repolarization (Phase 3) returns to the negatively charged resting phase (Phase 4). During the plateau phase of AP, the normal ST segment reflects no net voltage gradient in the myocardium. The QT interval in the ECG includes phases 0, 1, 2, and 3 of AP, indicating rapid depolarization and slow repolarization in myocardial cells [7]. SHS mainly comprises a slurring or notching J-point elevation, a downsloping ST-segment elevation, and a wide T-wave inversion, which can affect QT prolongation. Elevated J points in the form of slurring and notching are associated with a higher risk of sudden cardiac death (SCD) (relative risk 1.48–2.09). J-point elevations ≥0.2 mV in the inferior leads with a descending ST-segment variant have the highest SCD risk among various ST-segment elevation patterns (relative risk 3.14) [8]. Therefore, the presence of SHS is highly arrhythmogenic and markedly increases the likelihood of TdP [7].

The exact mechanism driving this ECG pattern remains unclear. It has been proposed that the SHS observed in thoracic and abdominal diseases is caused by two distinct mechanisms [9]. Reported cases have shown an association between intrathoracic pressure overload and SHS changes in the anterior leads and between intra-abdominal distention and SHS changes in the inferior leads. The manifestation of this ECG pattern in different leads, corresponding to the location of the affected cardiac tissue, is dependent on the opening and closing of ion channels involved in the rapid increase of intracavitary pressure. This mechanical mechanism, a pulsatile epidermal stretch attributed to an acute rise in intracavitary pressure, is responsible for the downsloping ST-segment elevation observed in thoracic or abdominal events [10]. In cardiac tissue, stretch-activated ion channels modulate cardiomyocyte conductivity under different stretch conditions, mainly intrathoracic and intra-abdominal distention. Additionally, the aforementioned mechanism can be related to electric alternans, a rarely observed cardiac phenomenon that often triggers malignant ventricular arrhythmias. Electric alternans refer to the alternation of the QRS complex axis, amplitude, or morphology, typically presenting as a 1-in-2 periodicity on the ECG.

The underlying electrophysiological mechanisms that lead to SHS-induced ventricular tachyarrhythmias are not fully understood. However, the currently leading hypotheses involve alterations in automaticity triggered activity, and phase 2 re-entry [11]. SHS, which mimics or suggests myocardial ischemia, arises due to electrical heterogeneities in the ventricular endocardium, midmyocardial cells, and epicardium during ventricular repolarization. The loss-of-function of L-type calcium current (ICa-L) or gain-of-function of adenosine triphosphate-dependent potassium current (IK-ATP) create transmural gradients, resulting in a descending ST-elevation pattern in the ECG. An imbalanced repolarization caused by a reduction in inward currents (late sodium current [INa-L] or ICa-L) or an increase in outward currents (transient outward potassium current [Ito], IK-ATP, or acetylcholine-dependent potassium current [IK-ACh]) leads to SHS. This process, in turn, causes integrated J-ST-T shifts, in which may involve the recruitment of IK-ATP channels. Although the ionic transfer of potassium from transmural myocardial injury is a fertile substrate for arrhythmogenesis, it is secondary to the myocardial oxygen supply-demand imbalance associated with a critical underlying condition.

There is evidence that SHS has a hereditary basis in the general population. Although the underlying mechanisms and triggering factors for SHS are not well defined, genetic mutations in cardiac ion channels or gap junctions are regarded as significant predisposing factors for SHS in critically ill patients. It is highly likely that electrical instability leading to life-threatening ventricular tachyarrhythmias primarily stems from genetically determined defects in the cardiac electrophysiological substrate. Previous studies have revealed that SHS is associated with *sodium channel protein type 5 subunit α* (*SCN5A)* and *potassium inwardly-rectifying channel, subfamily J, member 8 (KCNJ8)* missense mutations and could have an arrhythmogenic substrate in the inferior wall [12, 13, 14, 15]. Most importantly, SHS is mainly caused by a transmural voltage gradient resulting from an imbalanced ventricular repolarization between the endocardium and epicardium, which is associated with genetic susceptibility.

Increased sympathetic activity is believed to contribute to ventricular arrhythmias and SCD. In addition to increasing the heart rate, sympathetic hyperactivity can influence ventricular repolarization, leading to QT prolongation during intracerebral and subarachnoid hemorrhage (SAH) [16]. Sympathetic hyperactivity can cause hypokalemia, which further enhances the proarrhythmic potential of sympathetic hyperactivity [17]. For example, both prolonged QT intervals and macroscopic TWA have recently been reported in Takotsubo cardiomyopathy (TTC) [18]. Large negative T-waves and markedly prolonged QT intervals are frequent consequences of acute adrenergic stress [19]. Therefore, a consistent explanation confirmed that SHS was caused by adrenergically mediated QT prolongation due to sympathetic overstimulation in all serious pathophysiological disorders [20].

The development of SHS may be attributed to of sympathetic hyperactivity dysregulation. Changes in ion channels can be influenced by genetic predispositions and factors such as myocardial ischemia, hypoxia, acid-base imbalance, and/or electrolyte disturbances. Collectively these factors contribute to increased dispersion of ventricular repolarization. This leads to marked QT prolongation and macroscopic TWA, which predispose individuals to the manifestation of electrical instability and cause fatal ventricular arrhythmias [21].

## 3. Epidemiology

**Fig. 3.**
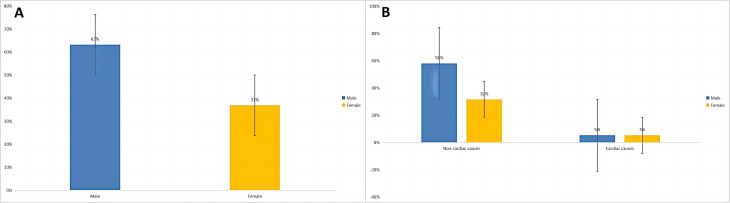
**Sex differences in selected patients with the spiked helmet sign in the electrocardiogram (A) and with noncardiac and cardiac causes (B)**.

Given the relatively recent identification, SHS findings remain limited to case reports and small case series studies. The true prevalence of SHS in critically ill patients remains unknown, and its actual distribution under various clinical conditions is less predictable. In the index case series, no significant difference was observed by sex, and the mean age of death was 53.83 ± 8.06 years in six selected patients with SHS on ECG. To date, Mahmoudi* et al*. [22] have conducted a systematic review of 39 case reports concerning the SHS, utilizing the preferred reporting items for systematic reviews and meta-analyses statements. Based on the same study flowchart, we conducted another systematic review and added two case reports written in the Hungarian language [23]. The study included a total 41 SHS patients, among whom 20 patients (59%; with a mean age of 61 ± 15 years) died (clinical outcomes were not reported for seven patients; Table 1). Among the included patients, there were more male deaths than female deaths. Furthermore, males with SHS on ECG had a higher likelihood of death due to noncardiac conditions compared to females (Fig. 3). Additionally, the mean age of those who died was higher than in previous reports. These findings suggest that age and sex may be factors associated with an increased risk of death in critically ill patients with SHS on ECG.

**Table 1.** **Clinical characteristics of selected patients who died with spiked helmet signs in the electrocardiogram**.

Case Number	Sex	Age (year)	Heart Rate (bpm)	Leads with SHS waveform	Long QT interval	Ventricular arrhythmia	Noncardiac causes				Cardiac causes	SHS presentation time (≤1 week)	In-hospital death (Time ≤1 week)	SHS resolution time (≤24 hours)	SHS resolution way
							Intrathoracic cause	Intra-abdominal cause	Intracranial cause	Others					
1	Male	46	NA	II, III	No	No	Yes	No	No	No	No	Yes	Yes	NA	NA
2	Female	54	130	II, III, aVF	No	No	No	No	No	Yes	No	No	Yes	NA	NA
3	Male	44	NA	II	No	No	No	No	No	Yes	No	Yes	No	NA	NA
4	Male	66	100	II, III, aVF	No	No	No	No	Yes	No	No	Yes	No	NA	NA
5	Female	55	NA	III, aVF	No	No	Yes	No	No	No	No	Yes	Yes	NA	NA
6	Female	58	130	II, III, aVF	NA	No	No	Yes	No	No	No	Yes	Yes	Yes	Spontaneous
7	Female	34	140	aVL, V1–6	No	No	Yes	No	No	No	No	Yes	No	Yes	Spontaneous
8	Male	84	78	II, III, aVF	Yes	No	No	No	No	No	Yes	Yes	Yes	Yes	Intervention
9	Male	60	110	V1–4	No	No	Yes	No	No	No	No	Yes	Yes	Yes	Intervention
10	Male	77	100	V2–5	Yes	Yes	Yes	No	No	No	No	Yes	Yes	Yes	Intervention
11	Male	54	55	II, V3–6	No	No	No	Yes	No	No	No	Yes	Yes	Yes	Spontaneous
12	Male	56	100	II, aVL, V1–3	NA	No	No	No	No	Yes	No	Yes	Yes	NA	NA
13	Male	72	95	V4–5	Yes	No	Yes	No	No	No	No	Yes	Yes	No	Spontaneous
14	Male	73	110	NA	Yes	No	Yes	No	No	No	No	Yes	Yes	NA	NA
15	Male	52	80	II, aVF	NA	No	No	Yes	No	No	No	Yes	Yes	Yes	Intervention
16	Female	70	185	V3–4	No	No	Yes	No	No	No	No	Yes	Yes	NA	NA
17	NA	55	130	II, III, aVF, V3–6	Yes	Yes	No	No	Yes	No	No	Yes	Yes	NA	Intervention
18	Female	40	100	I, aVL	Yes	No	No	No	Yes	No	No	Yes	Yes	NA	NA
19	Male	90	75	II, III, aVF	NA	No	No	No	No	Yes	No	Yes	Yes	Yes	Spontaneous
20	Female	73	98	V1–5	No	Yes	No	No	No	No	Yes	Yes	Yes	NA	NA

Bpm, beats per minute; NA, not applicable; SHS, spiked helmet sign.

## 4. Clinical Implications

**Fig. 4.**
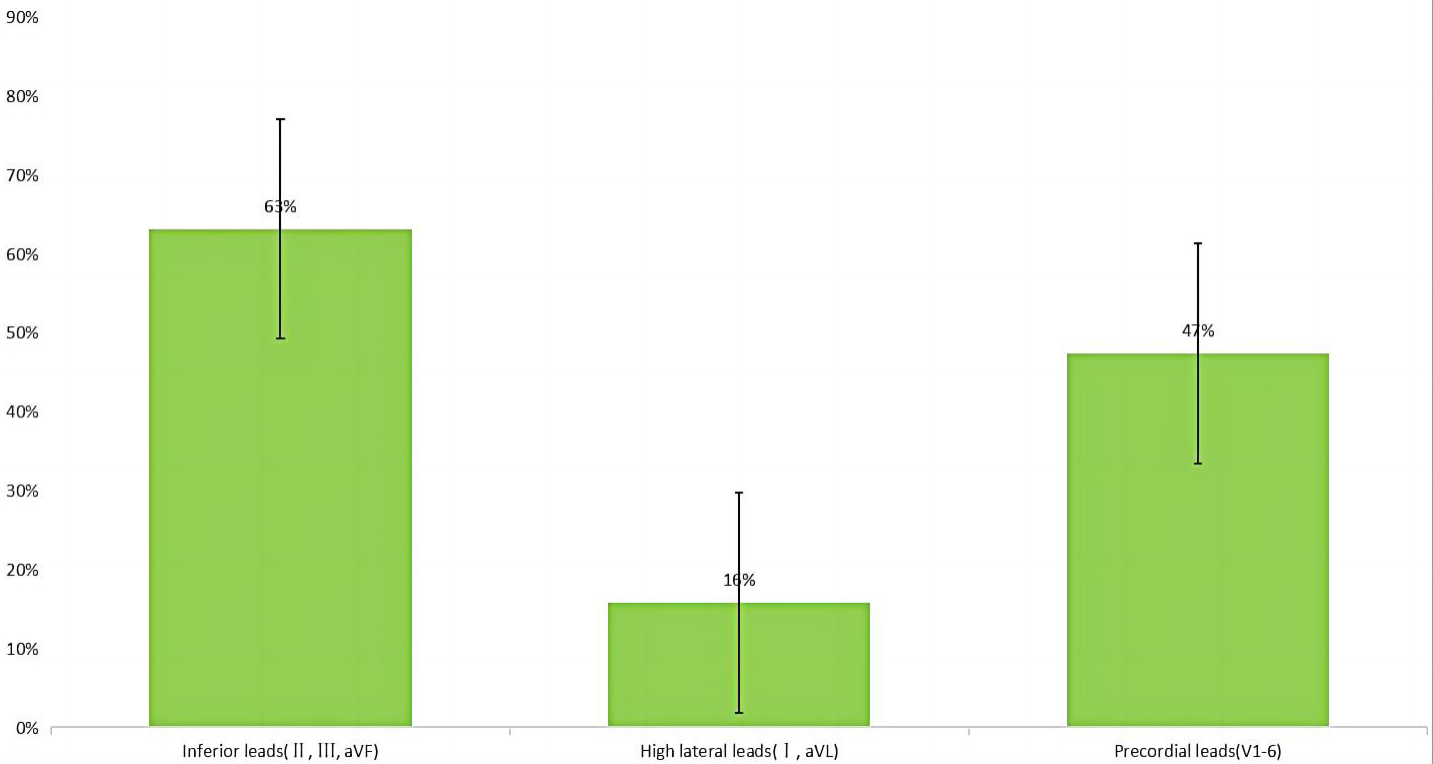
**Distribution of spiked helmet signs in electrocardiogram leads presented in a selected cohort of deceased patients**. This graph illustrates the distribution of spiked helmet signs in various electrocardiogram leads among a carefully chosen cohort of deceased patients. The leads are categorized into three groups: inferior leads, high lateral leads, and precordial leads. The presence and prevalence of spiked helmet signs are visually depicted, providing insights into the distribution patterns within the selected patient population.

**Fig. 5.**
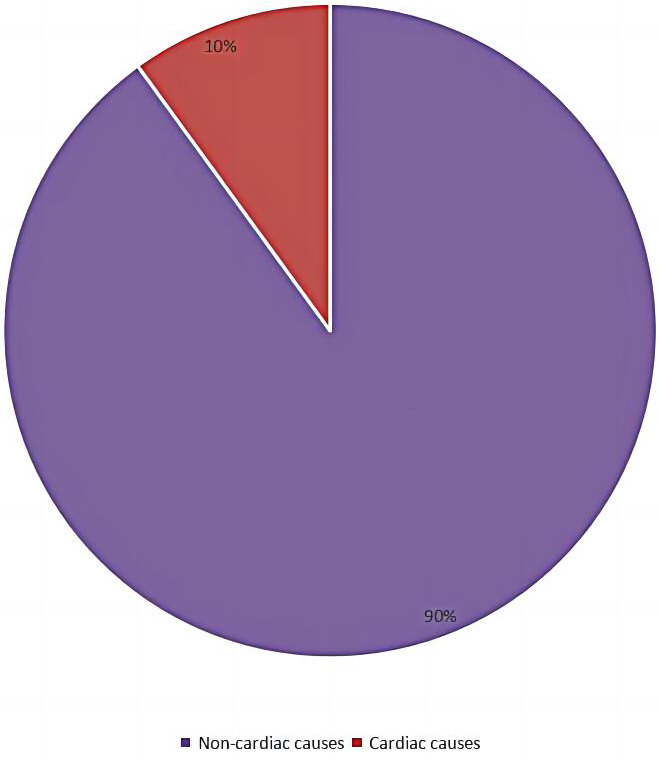
**Distribution of different causes in deceased SHS patients**. As illustrated above, non-cardiac causes are found most often in deceased SHS patient cohorts. SHS, spiked helmet sign.

The mean heart rate (HR) of the 17 patients who died was 107 ± 30 beats per minute (bpm) at the onset of SHS (ECGs were unavailable in three patients who died). Among the deceased patients, 11 (65%) had an elevated HR (≥100 bpm). The occurrence of SHS in the 20 patients who died was predominantly observed in the inferior leads (Fig. 4). These ECG features are commonly observed in critically ill patients with SHS. The search for distinctive ECG markers that indicate susceptibility to ventricular fibrillation (VF) and SCD is ongoing. SHS is associated with VF, a severe clinical course, and high mortality [24]. In our study, long QT intervals were present in six patients who died (37.5%), and ventricular arrhythmias were triggered in only three patients who died (15%). The occurrence of malignant arrhythmia following SHS was not commonly seen among those who died. Although often overlooked, SHS is associated with a high mortality rate in critically ill patients with noncardiac conditions [25]. A previous study reported that six (75%) of eight patients with SHS on ECG died of noncardiac causes. Our study also revealed that 18 (90%) of the 20 selected patients died of noncardiac causes (Fig. 5). SHS can occur in patients with acute ST-segment elevation myocardial infarction (STEMI), coronary vasospasm, or cardiac arrest [26, 27]. It is often observed in acute coronary artery occlusion [28]. However, SHS can be present in conditions other than primary cardiac diseases, such as right tension pneumothorax [29], pheochromocytoma, subarachnoid hemorrhage [30], or sepsis.

Identifying a correctable cause of the ECG can potentially improve the prognosis of right-sided tension pneumothorax. In a case study the presence of SHS with electric alternans was detected in a patient, this led to ventricular tachycardia due to the presence of a right tension pneumothorax, which was followed by cardiac arrest. In this case, if the clinician had identified the SHS earlier, the patient could have received a chest X-ray and echocardiography examination, potentially resulting in a timely diagnosis and prompt treatment [31].

Pheochromocytoma can present with various cardiovascular emergencies, including TTC. Excess catecholamine is central to the pathogenesis of pheochromocytoma. An accurate diagnosis is essential to managing pheochromocytoma, as it differs from acute coronary syndrome [32].

Acute central nervous system (CNS) events are most commonly related to SAH or Takotsubo syndrome [33], and are frequently associated with ECG abnormalities mimicking ST-segment elevation [34]. However, administering heparin and antiplatelet agents is detrimental to cerebral hemorrhage, and emergency coronary angiography can result in unnecessary delays in implementing appropriate therapeutic strategies. Therefore, recognizing SHS as an indicator of a potential CNS event can rectify misdiagnosis and potentially improve clinical outcomes [35].

Sepsis, a state of severe physical stress, can increase catecholamine levels, which activate the CNS and cause calcium overload in cardiomyocytes, leading to TTC [36]. Increasing evidence demonstrates that SHS is associated with an increased risk of VF and cardiogenic shock [37].

The clinical significance of SHS lies in its association with noncardiac conditions that carry a high risk of in-hospital mortality. However, evidence of this particular ECG abnormality is scarce. Previous reports supported the opinion that SHS was merely an optical illusion; therefore, it was not considered real [38]. Nevertheless, most researchers believe that SHS is not an artifact that can mimic ST elevation. Notably, different noncardiac acute conditions appear to be linked with the specific localization of SHS on the 12-lead ECG. When present in the inferior leads, it is usually the result of an acute abdominal event [39], such as gastrointestinal perforation. On the other hand, when present in the chest leads, it usually reflects an acute thoracic event [23], as demonstrated in the case of severe tension pneumothorax [40].

**Fig. 6.**
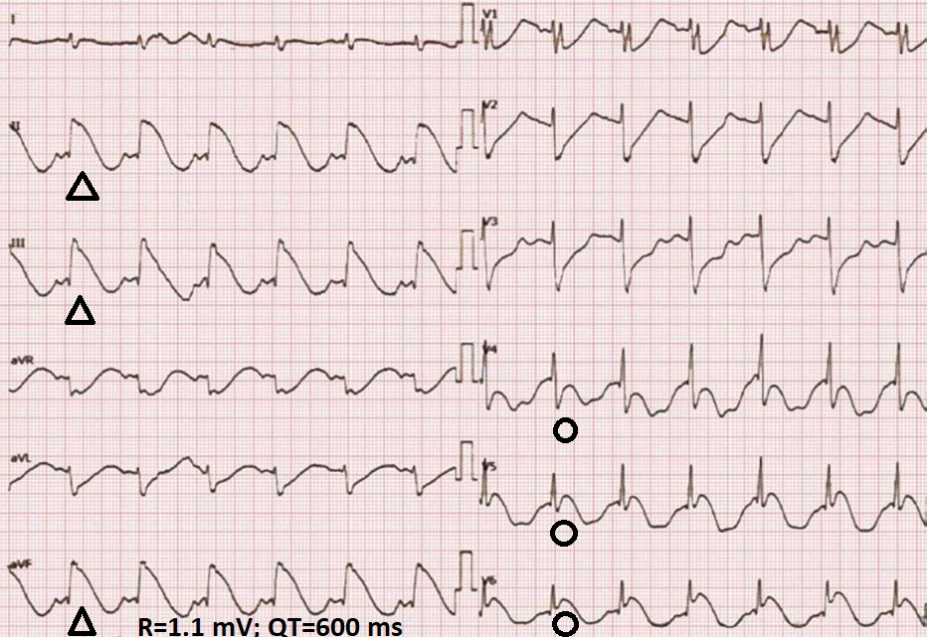
**Lambda-like (λ) pattern or shark-fin sign in the inferior (II, III, and aVF) leads and spiked helmet sign in the inferolateral (V4–6) leads in the electrocardiogram of an old man with Takotsubo cardiomyopathy**. The triangle mark indicates the lambda-like (λ) waveform or shark-fin sign, and the circular mark indicates the spiked helmet sign. (With permission from Takasaki *et al*. [18]).

The width and height of the elevated ST segment in SHS may be indicative of impending ventricular arrhythmias and SCD. The SHS width is an extreme manifestation of a prolonged QT(U) interval; however, whether the elevated J point can reach the peak of the QRS complex showing a shark-fin sign or lambda (λ) waveform requires further observation. As the disease progresses, the persistence of the SHS pattern is transient, and the lambda-like (λ) pattern or shark-fin sign may be the ultimate presentation of SHS in ECG (Fig. 6) [18]. A series of case reports indicate that these features may serve as novel risk predictors for life-threatening ventricular arrhythmias in ECG when they resemble a triangular configuration (**Supplementary Fig. 1**) [19, 37, 41]. A significant excess of adrenergic receptors is associated with long QT syndrome [42]. It is possible that some patients with SHS also have genetic abnormalities predisposing them to malignant ventricular arrhythmias, and critically ill patients with such conditions have a particularly high risk of developing VF. Large-scale studies, including multicenter and genetic assessments, are needed to clarify the potential value of this ECG pattern in identifying critically ill patients at high risk of sudden death.

ST-segment elevation in critically ill patients is a relatively common but nonspecific phenomenon, as most patients do not have acute STEMI. SHS is a unique electrocardiographic marker associated with high mortality in critically ill patients. When an apparent ST elevation takes the form of SHS, clinicians should actively search for a possible acute noncardiac pathology and note the absence of pathological Q-waves or symmetric T-wave inversion, which is typical of the ECG evolution after acute STEMI. Cardiac enzyme levels are not significantly elevated in patients with noncardiac illnesses. Although SHS can suggest a noncardiac event, clinicians should strongly consider the presence of STEMI if other clinical variables indicate this possibility. The presence of SHS has not been demonstrated to be either sensitive or specific for STEMI or non-STEMI diagnoses [43].

SHS is emerging as an underlying and frequently noncardiac condition that mimics a primary cardiac ECG abnormality characterized by a descending ST-segment elevation. In this study we found the SHS presentation time in 19 patients who died (95%) was less than 1 week after admission, and the in-hospital time in 17 patients who died (85%) was also less than 1 week after SHS presentation. Additionally, the SHS resolution time of eight deaths (89%) was less than 24 h, with five deaths (50%) experiencing spontaneous resolution. Therefore, the SHS presentation associated with impending death was transient and unpredictable. The development of this ominous ECG sign in critical illnesses should prompt an urgent reassessment. Greater awareness of SHS may prevent the unnecessary and potentially harmful initiation of an acute coronary syndrome protocol [44]. Recognizing this ECG pattern will guide physicians to prioritize comprehensive investigations, leading to an accurate diagnosis and the development of an appropriate treatment plan to resolve the underlying conditions, thereby normalizing the ST segments. Moreover, many antiarrhythmic drugs could be ineffective or even highly proarrhythmic in critically ill patients with SHS. In such cases, β-blockers may be considered the most suitable therapy. Large-scale studies are crucial to verifying the effectiveness of antiarrhythmic drugs in these patients.

## 5. Conclusions

SHS, a distinct electrocardiographic entity associated with imminent death, can manifest in critically ill patients in cardiac or noncardiac situations. In particular, a quick search for this ECG pattern plays a crucial role in recognizing and treating noncardiac conditions in older male patients.

## Abbreviations

AP, action potential; bpm, beats per minute; CNS, central nervous system; ECG, electrocardiogram; HR, heart rate; ICa-L, L-type calcium current; IK-ACh, acetylcholine-dependent potassium current; IK-ATP, adenosine triphosphate-dependent potassium current; INa-L, late sodium current; Ito, transient outward potassium current; SAH, subarachnoid hemorrhage; SCD, sudden cardiac death; SHS, spiked helmet sign; STEMI, ST-segment elevation myocardial infarction; TdP, Torsade de pointes; TTC, Takotsubo cardiomyopathy; TWA, T-wave alternans; VF, ventricular fibrillation.

## Supplementary Material

Supplementary material associated with this article can be found, in the online version, at https://doi.org/10.31083/j.rcm2409272.

## References

[1] Littmann L, Monroe MH (2011). The “spiked helmet” sign: a new electrocardiographic marker of critical illness and high risk of death. *Mayo Clinic Proceedings*.

[2] Simon A, Járai Z (2019). Is the spiked helmet sign the manifestation of long QT syndrome. *Journal of Electrocardiology*.

[3] Cardoso AF, Akamine MAV, Pessoa RM, Takitani ET, Kairiyama JV, Naritoni MK (2021). Spiked Helmet Sign: An Atypical Case of Transient ST-Segment Elevation on ECG. *Arquivos Brasileiros De Cardiologia*.

[4] Crinion D, Abdollah H, Baranchuk A (2020). An Ominous ECG Sign in Critical Care. *Circulation*.

[5] De Bernardi C, Halasz G, Cattaneo M (2020). Spiked Helmet Electrocardiographic Sign in a Patient with a Diagnosis of Thoracoabdominal Aortic Dissection. *JACC. Case Reports*.

[6] Oluyadi F, Kariyanna PT, Jayarangaiah A, Celenza-Salvatore J (2019). Helmet Sign on EKG: A rare Indicator of poor prognosis in critically ill patients. *American Journal of Case Reports*.

[7] Timour Q, Frassati D, Descotes J, Chevalier P, Christé G, Chahine M (2012). Sudden death of cardiac origin and psychotropic drugs. *Frontiers in Pharmacology*.

[8] Israel CW (2014). Mechanisms of sudden cardiac death. *Indian Heart Journal*.

[9] Tomcsányi J, Bózsik B (2022). Two forms of the spiked helmet sign are caused by two separate mechanisms. *Journal of Electrocardiology*.

[10] Reddy MJR, Johnson B, Garg J (2021). Spiked Helmet Sign. *The American Journal of Medicine*.

[11] Yan GX, Lankipalli RS, Burke JF, Musco S, Kowey PR (2003). Ventricular repolarization components on the electrocardiogram: cellular basis and clinical significance. *Journal of the American College of Cardiology*.

[12] Potet F, Mabo P, Le Coq G, Probst V, Schott JJ, Airaud F (2003). Novel brugada SCN5A mutation leading to ST segment elevation in the inferior or the right precordial leads. *Journal of Cardiovascular Electrophysiology*.

[13] Hu D, Viskin S, Oliva A, Carrier T, Cordeiro JM, Barajas-Martinez H (2007). Novel mutation in the SCN5A gene associated with arrhythmic storm development during acute myocardial infarction. *Heart Rhythm*.

[14] Guo Q, Ren L, Chen X, Hou C, Chu J, Pu J (2016). A novel mutation in the SCN5A gene contributes to arrhythmogenic characteristics of early repolarization syndrome. *International Journal of Molecular Medicine*.

[15] Haïssaguerre M, Chatel S, Sacher F, Weerasooriya R, Probst V, Loussouarn G (2009). Ventricular fibrillation with prominent early repolarization associated with a rare variant of KCNJ8/KATP channel. *Journal of Cardiovascular Electrophysiology*.

[16] Tomcsányi J, Bózsik B, Tomcsányi K (2019). Spiked helmet electrocardiographic sign in a patient with intracerebral haemorrhage. *Acta Cardiologica*.

[17] Hasanien AA, Drew BJ, Howie-Esquivel J (2014). Prevalence and prognostic significance of long QT interval in patients with acute coronary syndrome: review of the literature. *The Journal of Cardiovascular Nursing*.

[18] Takasaki A, Nakamori S, Dohi K (2019). Massive ST-Segment Elevation and QTc Prolongation in the Emergency Department. *Circulation*.

[19] Tarantino N, Santoro F, Guastafierro F, Di Martino LFM, Scarcia M, Ieva R (2018). “Lambda-wave” ST-elevation is associated with severe prognosis in stress (takotsubo) cardiomyopathy. *Annals of Noninvasive Electrocardiology: the Official Journal of the International Society for Holter and Noninvasive Electrocardiology, Inc*.

[20] Bezgin T, Çelik Aİ, Çağdaş M (2021). A confusing mimicker of ST-segment elevation myocardial infarction: spiked helmet sign. *Acta Cardiologica*.

[21] Madias JE (2019). Towards a resolution of the mechanism of “spiked helmet ECG sign” in takotsubo syndrome and other acute life-threatening illnesses. *Journal of Electrocardiology*.

[22] Mahmoudi E, Hui JMH, Leung KSK, Satti DI, Lee YHA, Li KHC (2023). Spiked Helmet Electrocardiographic Sign-A Systematic Review of Case Reports. *Current Problems in Cardiology*.

[23] Tomcsányi J, Frész T (2013). Spiked helmet sign ST-segment elevation. *Orvosi Hetlap*.

[24] Madias JE (2018). “Spiked Helmet” electrocardiogram sign in a patient with takotsubo syndrome: Similarities with a previously described marker. *The American Journal of Emergency Medicine*.

[25] Agarwal A, Janz TG, Garikipati NV (2014). Spiked helmet sign: An under-recognized electrocardiogram finding in critically ill patients. *Indian Journal of Critical Care Medicine: Peer-reviewed, Official Publication of Indian Society of Critical Care Medicine*.

[26] Alper AT, Tekkesin AI, Çinier G, Turkkan C, Baranchuk A (2017). First description of a Brugada phenocopy in the inferior leads in the context of an acute inferior myocardial infarction. *Europace: European Pacing, Arrhythmias, and Cardiac Electrophysiology: Journal of the Working Groups on Cardiac Pacing, Arrhythmias, and Cardiac Cellular Electrophysiology of the European Society of Cardiology*.

[27] Yu M, Zhang Q, Huang X (2018). Acute coronary syndrome due to right coronary spasm and documented lambda-like J waves. *Clinical Research in Cardiology: Official Journal of the German Cardiac Society*.

[28] Minotti B, Scheler J, Sieber R, Scheler E (2021). The “Spiked Helmet” Sign Associated with ST-Elevation Myocardial Infarction: A Case Report. *Clinical Practice and Cases in Emergency Medicine*.

[29] Abdelghany M, Chaudhary A, Liu K (2017). Chest Pain in an 18-Year-Old Man. *Circulation*.

[30] Hamade H, Jabri A, Yusaf A, Nasser MF, Karim S (2021). The Spiked Helmet Sign: A Concerning Electrocardiographic Finding. *JACC. Case Reports*.

[31] Robert J, Derkenne C, Jost D, Tourtier JP (2017). Out-of-Hospital Cardiac Arrest: An Underlying Reversible Cause. *Circulation*.

[32] Bhasin D, Isser HS, Gupta A (2021). Chest Pain with ST-Segment Elevation in a Young Woman: A Broken Heart. *Circulation*.

[33] Hankovszky P, Tömösvári A, Hawchar F, Farkas T, Rudas L (2021). Tachycardia dependent early repolarisation pattern in subarachnoid haemorrhage related takotsubo syndrome. *Journal of Electrocardiology*.

[34] Laundon RK, Littmann L (2019). Spiked helmet pattern ST elevation in subarachnoid hemorrhage. *Journal of Electrocardiology*.

[35] Shih SY, Hou YT, Lin PC, Chen YL, Chien DS, Yiang GT (2021). The Spiked Helmet Sign Predicting a Poor Outcome in a Patient with Non-Myocardial Infarction ST-Segment Elevation. *Medicina (Kaunas, Lithuania)*.

[36] Samadov F, Gasimov E, Aliyev F, Isayev E (2018). The “Spiked Helmet” sign - A potential relationship to Takotsubo cardiomyopathy. *The American Journal of Emergency Medicine*.

[37] Zhang B, Yin ZW, Chen W (2022). Shark Fin Electrocardiogram in the Intensive Care Unit. *Circulation*.

[38] Littmann L (2019). The electrocardiographic spiked helmet sign: Is it real, artifact, or optical illusion. *Journal of Electrocardiology*.

[39] Cisewski DH, Madias JE, Wong L (2019). Utilization of the Electrocardiographic “Spiked Helmet” Sign in the Diagnosis of Intra-Abdominal Pathology Within the Emergency Setting. *The Journal of Emergency Medicine*.

[40] Littmann L, Proctor P (2014). Real time recognition of the electrocardiographic “spiked helmet” sign in a critically ill patient with pneumothorax. *International Journal of Cardiology*.

[41] Riera ARP, Ferreira C, Schapachnik E, Sanches PC, Moffa PJ (2004). Brugada syndrome with atypical ECG: downsloping ST-segment elevation in inferior leads. *Journal of Electrocardiology*.

[42] Aliyev F, Abdulkerimov V, Gul EE, Samedov F, Isayev E, Ferecov E (2017). Spiked helmet sign after percutaneous left stellate ganglion ablation in a patient with long QT syndrome. *Journal of Electrocardiology*.

[43] Tomcsányi J, Frész T, Proctor P, Littmann L (2015). Emergence and resolution of the electrocardiographic spiked helmet sign in acute noncardiac conditions. *The American Journal of Emergency Medicine*.

[44] Lin YK, Chen KC, Huang YN, Chang H (2022). The ‘spiked-helmet’ sign in patients with myocardial injury. *Journal of Electrocardiology*.

