# Supplementary material for: Spiked Helmet Sign: An Uncommon Electrocardiographic Marker
Source: Rev Cardiovasc Med. 2023 Sep 25;24(9):272. doi: 10.31083/j.rcm2409272 (PMC11270090; doi:10.31083/j.rcm2409272)
Supplement: Supplementary file 1 [file 2153-8174-24-9-272-s1.zip › 2153-8174-24-9-272-s1.docx]

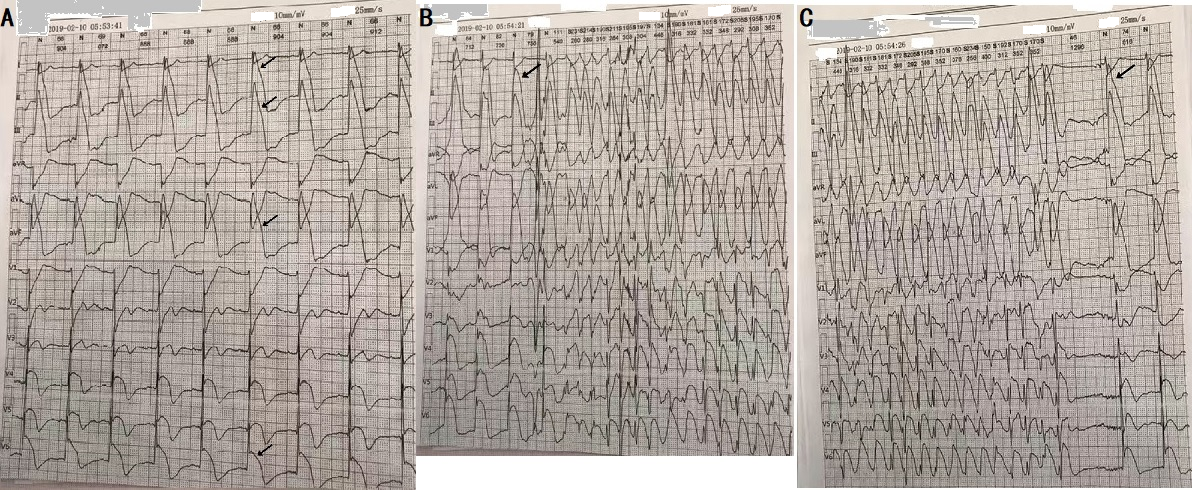


**Supplementary Fig. 1.** The lambda-like (λ) pattern or shark-fin sign (indicated by the black arrows) in the inferior (Ⅱ, Ⅲ, aVF) leads and the spiked helmet sign in the inferolateral (V4-6) leads (A), followed by a paroxysmal polymorphic ventricular tachycardia (B and C) detected by dynamic electrocardiogram.
